# The causal relationship between gut microbiota and diabetic neuropathy: a bi-directional two-sample Mendelian randomization study

**DOI:** 10.3389/fendo.2024.1402014

**Published:** 2024-07-10

**Authors:** Long Xie, Wen Gan, GuangRong Cai

**Affiliations:** ^1^ Department of Orthopedics, The Fourth Hospital of Changsha (The Changsha Affiliated Hospital of Hunan Normal University), Hunan Normal University, Changsha, China; ^2^ Department of Thoracic Surgery, Yuebei People’s Hospital, Shaoguan, Guangdong, China; ^3^ Trauma Department of Orthopaedics, Yuebei People’s Hospital, Shaoguan, Guangdong, China

**Keywords:** gut microbiota, diabetic neuropathy, Mendelian randomization, causal relationship, bi-directional

## Abstract

**Background:**

Many studies suggest a strong correlation between gut microbiota (GM) and diabetic neuropathy (DN). However, the precise causal relationship between GM and DN has yet to be fully elucidated. Hence, a bi-directional Mendelian randomization (MR) analysis was used to examine the association between GM and DN.

**Methods:**

Widely known genome-wide association study (GWAS) of GM was collected from the MiBio Gen project. Summary-level datasets for DN were taken from the FinnGen project. Inverse variance weighted approach was used for evaluating the causal relationship between GM and DN. Subsequently, pleiotropy and heterogeneity tests were performed to verify the reliability of the data. Furthermore, a bidirectional two-sample MR analysis was done to investigate the directionality of the causal relationships. Gene Ontology analysis was conducted to identify the associations that could indicate biological functions.

**Results:**

We identified potential causal associations between GM and DN (*p*< 0.05 in all three MR methods). Among them, we found increased levels of Christensenellaceae R-7 (Odds ratio, OR= 1.52; 95% confidence interval, CI = 1.03–2.23; *p* = 0.03), Ruminococcaceae UCG013 (OR =1.35; 95% CI = 1.00–1.85; *p* = 0.04), and *Eggerthella* groups (OR = 1.27; 95% CI = 1.05–1.55; *p* = 0.01), which may be associated with a higher risk of DN, while increased levels of Peptococcaceae (OR = 0.69; 95% CI = 0.54–0.90; *p*< 0.01) and *Eubacterium coprostanoligenes* groups (OR = 0.68; 95% CI = 0.49–0.93; *p* = 0.01) could be associated with a lower risk. Gene Ontology pathway analysis revealed enrichment of genes regulated by the associated single-nucleotide polymorphisms (SNPs) in the apical plasma membrane, glycosyltransferase activity, hexosyltransferase activity and membrane raft. Reverse MR analyses indicated that DN was associated with five microbial taxa in all three MR methods.

**Conclusion:**

The results of our study validate the possible causative relationship between GM and DN. This discovery gives new perspectives into the mechanism on how GM influences DN, and establishes a theoretical foundation for future investigations into targeted preventive measures.

## 1 Introduction

Diabetic neuropathy (DN) is a common and burdensome complication of diabetes that is significant but often over-looked. It can markedly impair psychological functions and quality of life (1). Diabetic Peripheral Neuropathy (DPN) is the most frequently observed type of DN that affects the feet and legs. It presents a range of symptoms, including pain, numbness, and severe discomfort. autonomic dysfunction, however, affects the autonomic nervous system that regulates involuntary bodily functions. This dysfunction contributes to various problems, such as cardiovascular dysfunction characterized by blood pressure and heart rate changes, gastrointestinal dysfunction leading to gastroparesis, and urogenital dysfunction affecting bladder control and sexual function (2). DPN alone contributes to more than $10 billion in annual healthcare expenses, exceeding one-fourth of the total direct medical costs of diabetes (3). Managing DN requires a comprehensive approach that includes strict glycemic control to slow neuropathy progression, pain management and treatment of autonomic symptoms to enhance quality of life (4). Early diagnosis and thorough management are key to prevent complications and improve patient outcomes. Due to the limited treatment options for DN, it is crucial to investigate and identify new therapeutic targets (5).

Gut microbiota (GM) generally refers to the bacteria residing in the human gut. It plays an important role in regulating a wide array of physiological functions in the host and providing protection against pathogenic bacteria (6). GM is involved in processes such as digestion, immune system modulation, also influencing mood and behavior through the gut–brain axis (7, 8). The pathogenesis of microbiota dysbiosis significantly contributes to the onset and advancement of diabetes mellitus and its complications, such as cardiovascular disease, nephropathy, and DN, by promoting systemic inflammation and disrupting metabolic functions (9). However, these observational studies did not show a causal relationship between GM with DN, and it is still uncertain whether reverse causality weakens this correlation.

Mendelian randomization (MR) is a method in genetic epidemiology that uses genetic variants as instrumental variables (IVs) to assess the causal relationship between an exposure and outcome (10). Genome-wide association studies (GWASs) offer extensive datasets featuring many single nucleotide polymorphisms (SNPs) and significant sample sizes. MR leverages Mendelian inheritance laws using one or more genetic polymorphisms as the exposure variable. This makes GWAS-based MR a compelling method for determining causality (11, 12).

The two-sample MR technique offers increased statistical power to identify the causal effects between exposure factors and outcomes using published summary estimates from various large-scale GWASs (13). Also, large-scale summary statistics enable the analysis of the relationships between GM and DN by enhancing the statistical power of two-sample MR analysis. Hence, a bidirectional MR methodology was employed to investigate the potential causal association between GM and DN by combining data from the MiBioGen and FinnGen consortiums’ GWASs on genetic variations. The adoption of a bidirectional MR strategy enhanced the robustness of our findings against potential confounding variables and reverse causation. Finally, we conducted a gene ontology (GO) analysis using lead SNPs to investigate the GM’s biological impact on DN. This GO analysis offered deeper understanding of the physiological mechanisms involved. Our research opens new avenues and provides fresh insights for future DN studies.

## 2 Methods

### 2.1 Study design

**Figure 1:**
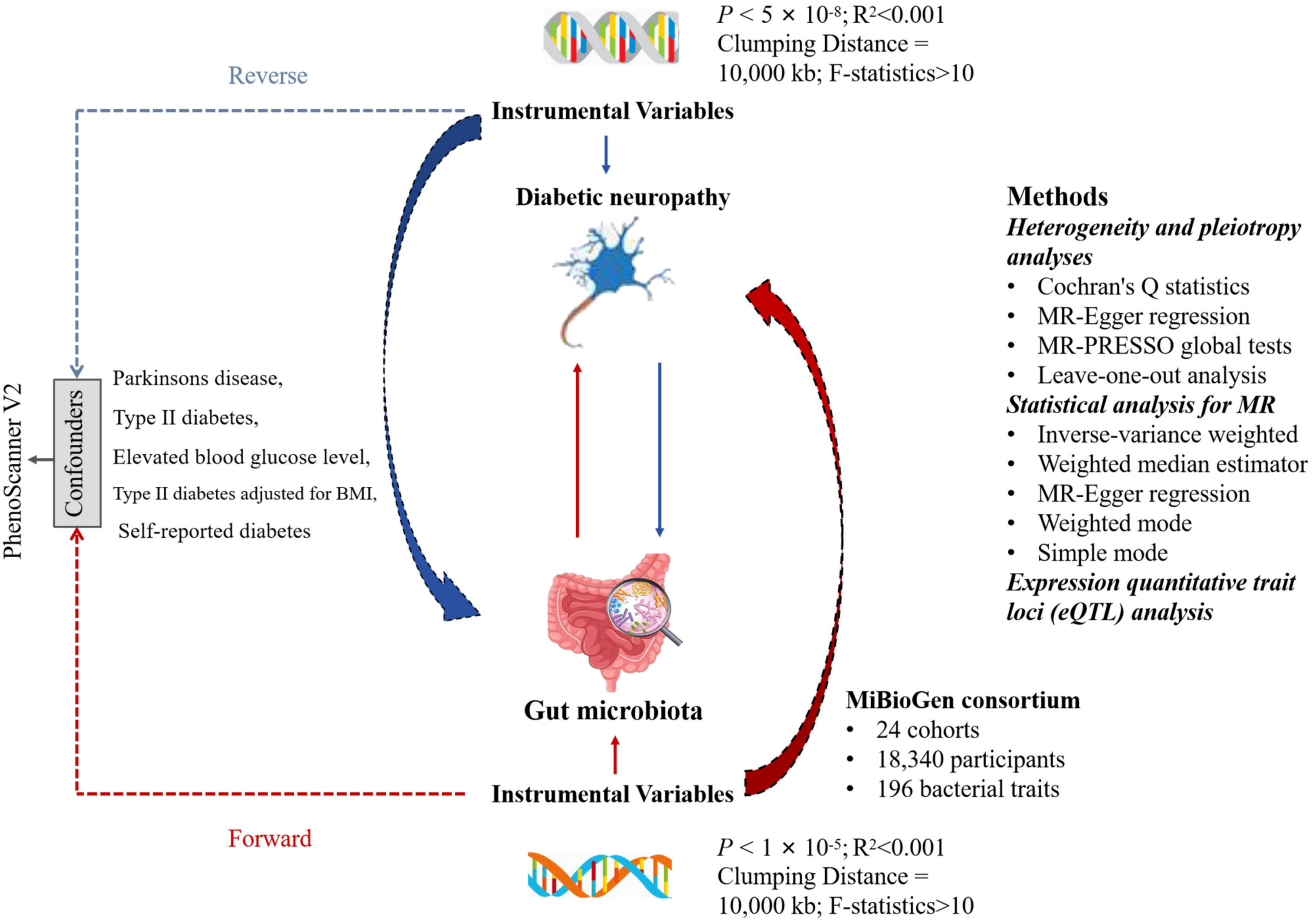
The flowchart for the study of the association between gut microbiota and Diabetic neuropathy. LD, linkage disequilibrium; SNP, single nucleotide polymorphism.

To establish the potential causal relationships between GM and DN, we employed a bi-directional MR analysis, which provides stronger associations by minimizing the biases present in the traditional epidemiologic observational studies. The flow chart of the study design is shown in **Figure 1**. To perform the study, it is necessary that three fundamental assumptions are satisfied: (1) A strong correlation between IVs and exposure; (2) No correlation between IVs and confounders; and (3) IVs can only affect the outcomes through exposure (14). IVs that fulfill these three assumptions were included in this MR study. Our results were reported according to the STROBE-MR guidelines (15).

### 2.2 Data source of exposure and outcome

This was a multi-ethnic large-scale GWAS that coordinated 16S ribosomal RNA gene sequencing profiles and genotyping data from 18,340 participants of 24 cohorts from the USA, Canada, Israel, South Korea, Germany, Denmark, Netherlands, Belgium, Sweden, Finland, and UK to explore the association between the autosomal human genetic variants and GM (16). A total of 211 bacterial traits (classified into specific phylum, class, order, family, and genus) were obtained, and the sample size was 14,306. Out of the 211 traits selected, 15 bacterial traits did not have specific species names. Hence, we excluded them and used the remaining 196 traits for analysis. All the original studies were approved ethically and participants’ consents were obtained. In this study, the GWASs and associated datasets were shown in **Table 1**.

**Table 1:** The present study used genome-wide association studies (GWAS) and associated datasets to conduct our analysis.

Exposure or outcome	Sample size	Ancestry	Links for data download	PMID
Human gut microbiome	18,340 participants	Mixed	https://mibiogen.gcc.rug.nl	33462485
Diabetic neuropathy	1,415 cases, 162,201 controls	European	https://gwas.mrcieu.ac.uk/datasets/finn-b-DM_NEUROPATHY/	–

### 2.3 Instrumental variables

IVs were chosen from a GWAS dataset provided by the international consortium MiBio Gen. These IVs are specifically associated with the makeup of the human GM. First, consistent with prior MR studies, we identified significant SNPs for the respective GM using a cut-off value of *p<* 1×10^−5^ (17). When conducting a reverse MR analysis with DN as the exposure, we set the threshold at *p<* 5×10^-8^ for selecting SNPs. Second, the clump program in PLINK software was adopted to exclude the dependent IVs of R^2^< 0.001 (clumping window size = 10,000 kb), which were obtained using the 1000 Genome Projects reference panel in Europe (18). Third, an important step in MR is to ensure that the effects of the SNPs on the exposure correspond to the same allele as that on the outcome. To avoid distortion of strand orientation or allele coding, we removed palindromic SNPs (such as, with A/T or G/C alleles). To assess the presence of weak instrument bias, the F-statistic for the IVs was computed using the formula $F=\frac{R^{2}\left(N-1-K\right)}{\left(1-R^{2}\right)K}$ , where R^2^ is the proportion of variance in the exposure explained by the genetic variants, N is the sample size, and K is the number of instruments (19). A weak instrument, indicated by an F-value below 10, was excluded (20). Additionally, by searching for pleiotropic SNPs of confounders in PhenoScanner V2 (21), we eliminated certain IVs that were significantly associated with potential confounders (*p<* 1×10^−5^). When the exposure was GM, potential confounders included Parkinsons disease, type II diabetes mellitus, elevated blood glucose level, type II diabetes adjusted for body mass index (BMI), and self-reported diabetes. In reverse MR analysis with DN as the exposure, no potential confounders were identified. The remaining IVs were then used for subsequent MR analysis.

### 2.4 Heterogeneity and pleiotropy analyses

We conducted a heterogeneity test utilizing Cochran’s Q statistics. A *p<* 0.05 indicated significant heterogeneity (10). Horizontal pleiotropy, which implies that IVs influence outcomes through paths other than the causal effects, can potentially lead to false-positive results (*p<* 0.05) (22). To evaluate the direct relationship between the chosen IVs and outcome, horizontal pleiotropy was tested using MR-Egger intercept test and MR-PRESSO global tests. Significant outliers identified in the MR-PRESSO analysis were excluded to reduce the influence of horizontal pleiotropy (23). Furthermore, a leave-one-out analysis was conducted to validate the robustness of the results (24).

### 2.5 Statistical analysis for MR

For the MR analysis, we employed five methods: the inverse-variance weighted (IVW) test, weighted median estimator (WME), MR-Egger regression, weighted mode (WMe) and simple mode (SM). IVW was the primary method, complemented by the other four methods (25). All the statistical analyses were conducted using R programming, version 4.2.3 (R Foundation for Statistical Computing, Vienna, Austria). For MR analyses, we utilized the “Two sample MR” (version 0.5.7) and “MR_PRESSO” (version 1.0) R packages (23).

### 2.6 Gene ontology enrichment analysis

To examine the function of IVs in mediating causality between exposure factors and outcomes, we utilized IV SNPs derived from MR analysis. By integrating these SNPs with the data from the eQTLGen database, we analyzed the genes regulating gene expression from the cis-expression quantitative trait loci (cis-eQTL) standpoint and nearest gene method (26). Using the R package “ClusterProfiler” we conducted a gene ontology (GO) enrichment analysis on these genes to investigate the patterns of gene expression regulation (27).

## 3 Results

### 3.1 Selection of IVs

To analyze the effects of GM on DN, we selected 2–12 SNPs for GM species as IVs. Some analyses were unsuccessful due to the absence of SNPs following harmonization. The F statistics for IVs indicated that the estimates were less likely to suffer from weak instrumental bias (F > 10, **Supplementary Table 1**).

### 3.2 Potential causal associations between the GM and DN

**Figure 2:**
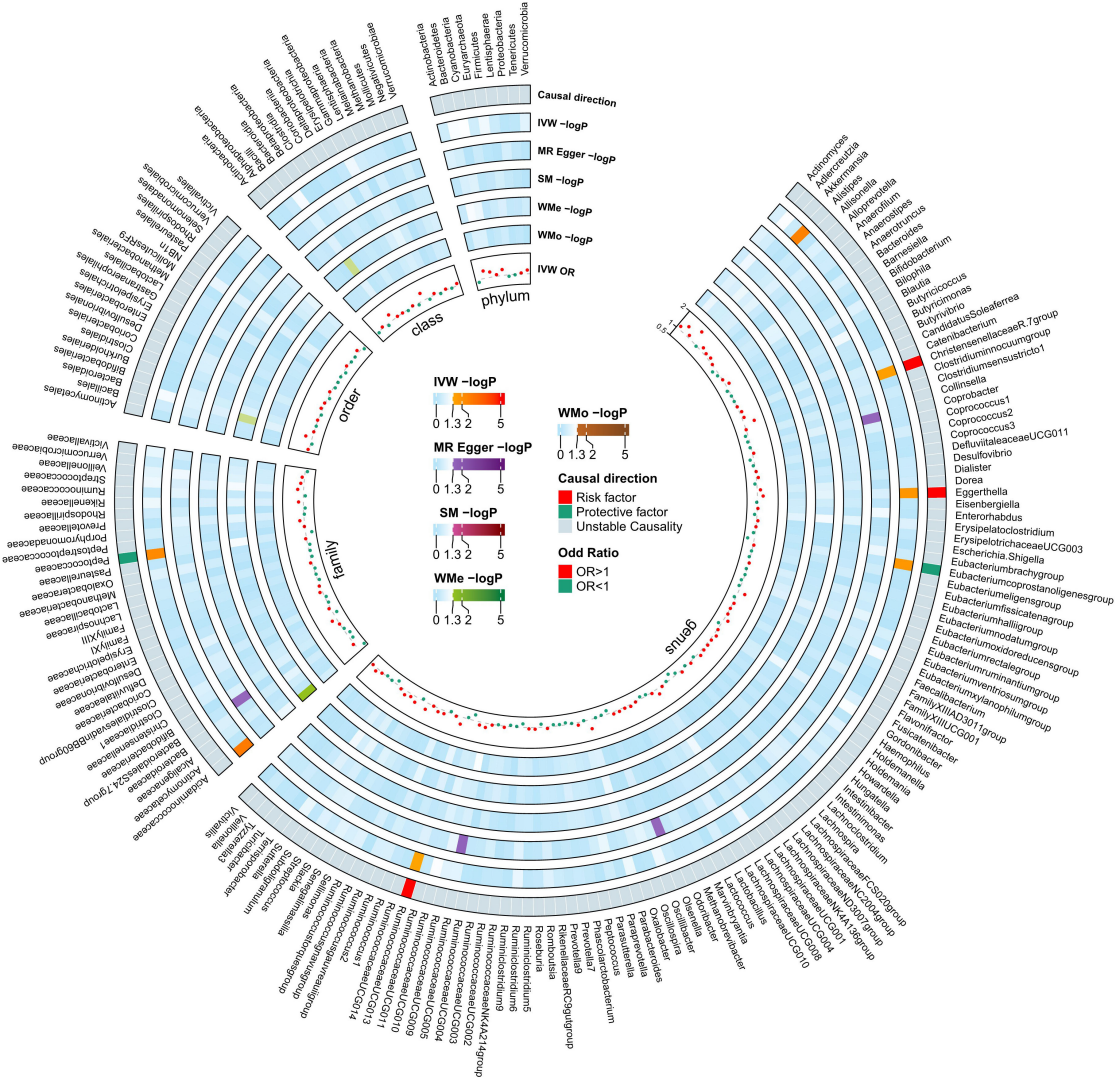
Mendelian Randomization analyses illustrating the causal effect of the gut microbiome on diabetic neuropathy. In both circular heatmaps, the data layers, from inside to out, represent the odds ratios calculated using the Inverse Variance Weighted (IVW) method, followed by −log10(p values) for IVW, Weighted Median (WMo), Weighted Mode (WMe), Simple Median (SM), and MR-Egger methods, respectively. The outermost ring illustrates the acceptance of effect direction as determined by the five MR methodologies: IVW (p< 0.05), MR-Egger, SM, WMe, and WMo. IVW, inverse variance weighted; SM, simple mode; WMe, weighted median; WMo: weighted mode; MR, mendelian randomization.

**Figure 3:**
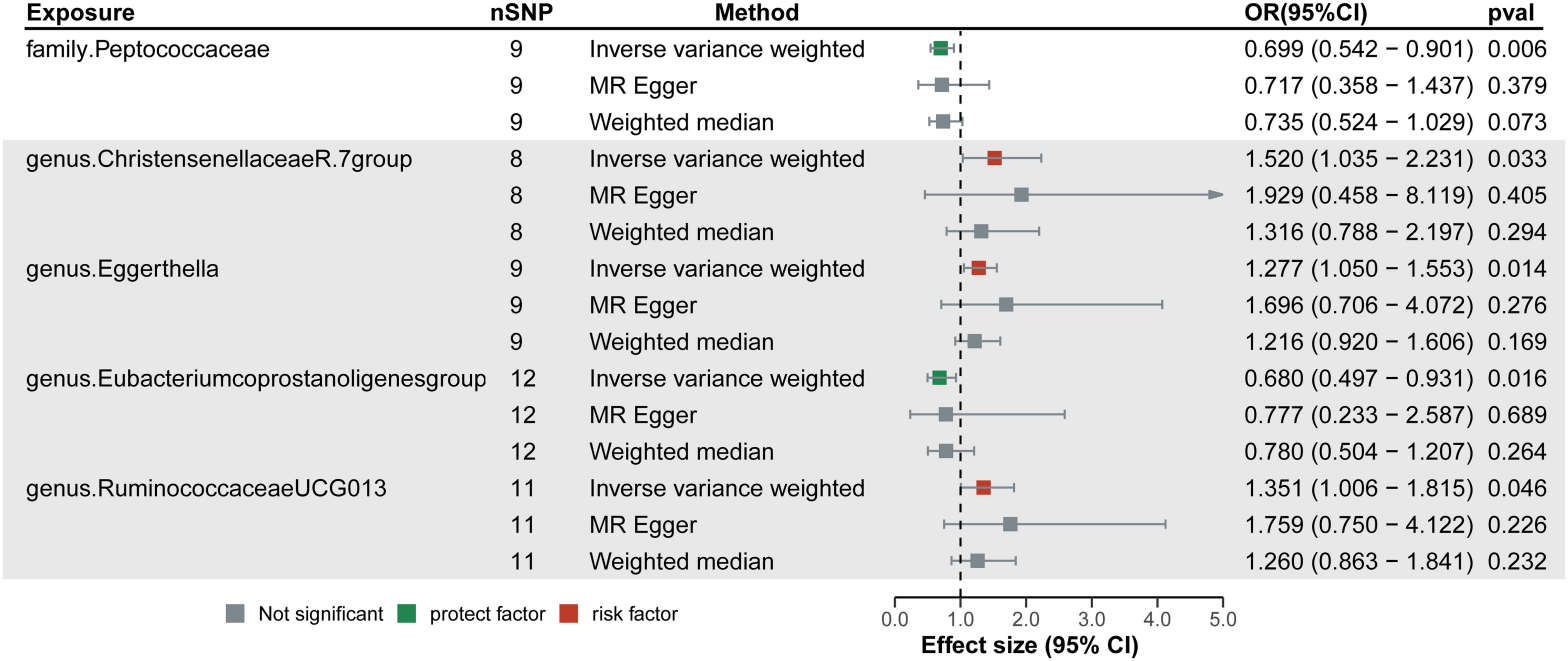
Forest plots of the significant causal effect of gut microbiota on diabetic neuropathy were calculated using the inverse variance weighted method, MR-Egger, and weighted median. The forest plots demonstrate that elevated levels of Christensenellaceae R-7, Ruminococcaceae UCG-013, and *Eggerthella* groups were risk factors for of diabetic neuropathy. While higher levels of Peptococcaceae and *Eubacterium coprostanoligenes* groups were protective factors. IVW, Inverse variance weighted.

As seen in **Figure 2**, in both circular heatmaps, the data layers, from inside to out, represent the odds ratios (OR) calculated using the IVW method, followed by −log10 (*p* values) for IVW, Weighted Median (WMo), WMe, SM, and MR-Egger methods, respectively. The outermost ring illustrates the agreement of effect direction as determined by the five MR methodologies: IVW (*p*< 0.05), MR-Egger, SM, WMe, and WMo. We identified three risk factors (genus Christensenellaceae R-7group, *Eggerthella*, and Ruminococcaceae UCG-013) and two protecting factors (family. Peptococcaceae and *Eubacterium coprostanoligenes* group) related to DN after setting a standard in which the IVW method demonstrated a significant difference (p< 0.05), and the five methods indicated consistent directions. Details and statistics are given in **Figure 3**. Specifically, we observed elevated levels of Christensenellaceae R-7 (OR = 1.52; 95% confidence interval, CI = 1.03–2.23; *p* = 0.03), Ruminococcaceae UCG-013 (OR = 1.35; 95% CI = 1.00–1.85; *p* = 0.04), and *Eggerthella* groups (OR = 1.27; 95% CI = 1.05–1.55; *p* = 0.01), which may be linked to an increased risk of DN. Conversely, higher levels of Peptococcaceae (OR = 0.69; 95% CI = 0.54–0.90; *p<* 0.01) and *Eubacterium coprostanoligenes* groups (OR = 0.68; 95% CI = 0.49–0.93; *p* = 0.01) could indicate a reduced risk of DN (**Supplementary Table 2**). The leave-one-out investigation revealed that removing any of the SNPs did not affect the overall results, suggesting that this MR analysis is extremely robust (**Supplementary Figure 1**).

### 3.3 Sensitivity analyses

The MR-Egger, WMe, SM, and WMo techniques showed comparable causal estimates for size and direction. We discovered no evidence of horizontal pleiotropy for GM in DN with *p* > 0.05 when utilizing the MR-Egger regression intercept method. MR-PRESSO analysis indicated no outliers in the findings. In addition, the findings of the Cochrane’s Q statistics indicated no substantial heterogeneity (*p* > 0.05) (**Supplementary Table 3**). Scatter plots were utilized to assess the MR models and show the intercept of the MR-Egger slope (**Supplementary Figure 2**).

### 3.4 Reverse MR analysis

**Figure 4:**
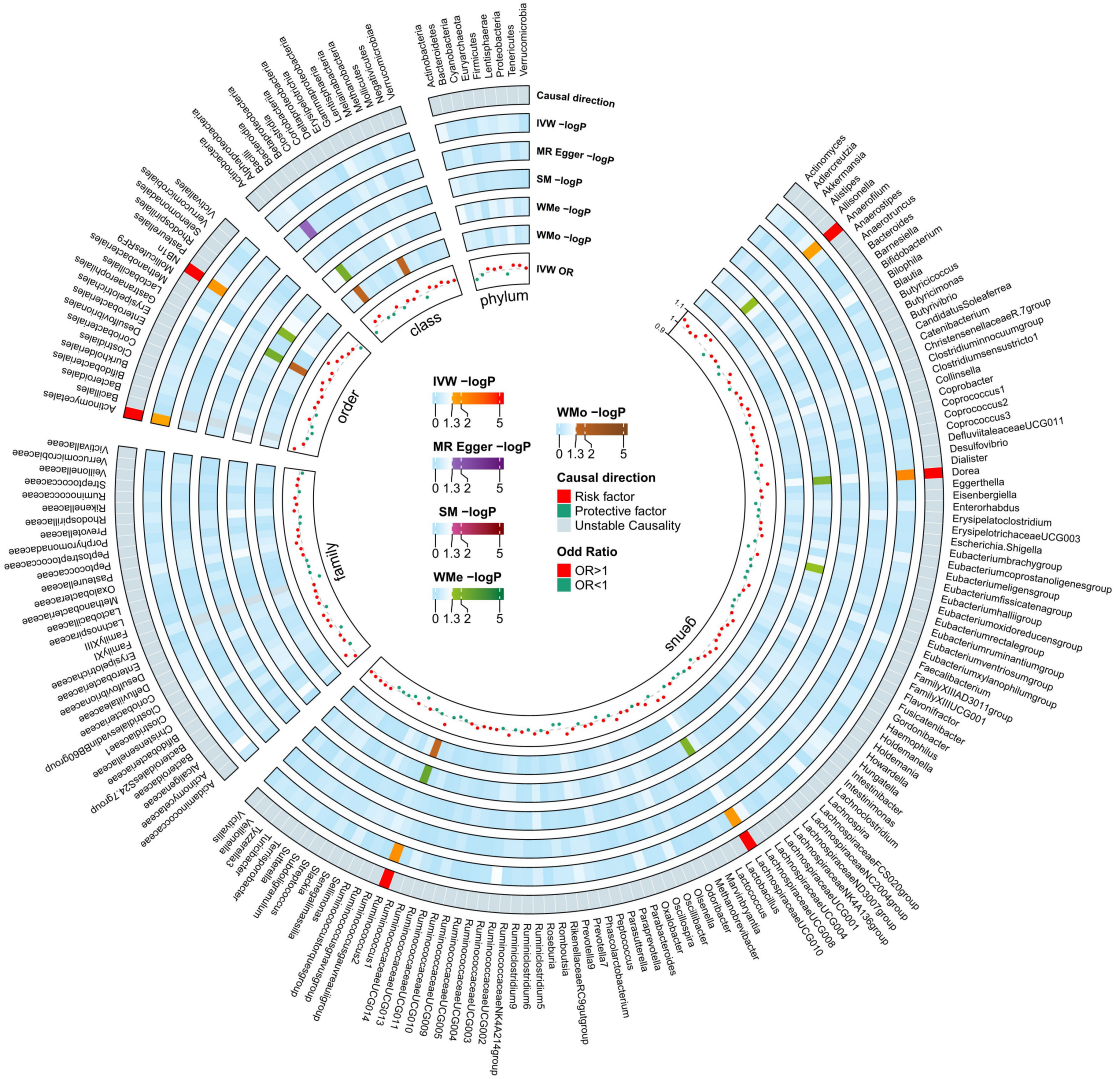
Mendelian Randomization analyses illustrating the causal effect of the diabetic neuropathy on gut microbiome. In both circular heatmaps, the data layers, from inside to out, represent the odds ratios calculated using the Inverse Variance Weighted (IVW) method, followed by −log10(*p* values) for IVW, Weighted Median (WMo), Weighted Mode (WMe), Simple Median (SM), and MR-Egger methods, respectively. The outermost ring illustrates the concordance of effect direction as determined by five MR methodologies: IVW (*p*< 0.05), MR-Egger, SM, WMe, and WMo. IVW, inverse variance weighted; SM, simple mode; WMe, weighted median; WMo: weighted mode; MR, mendelian randomization.

**Figure 5:**
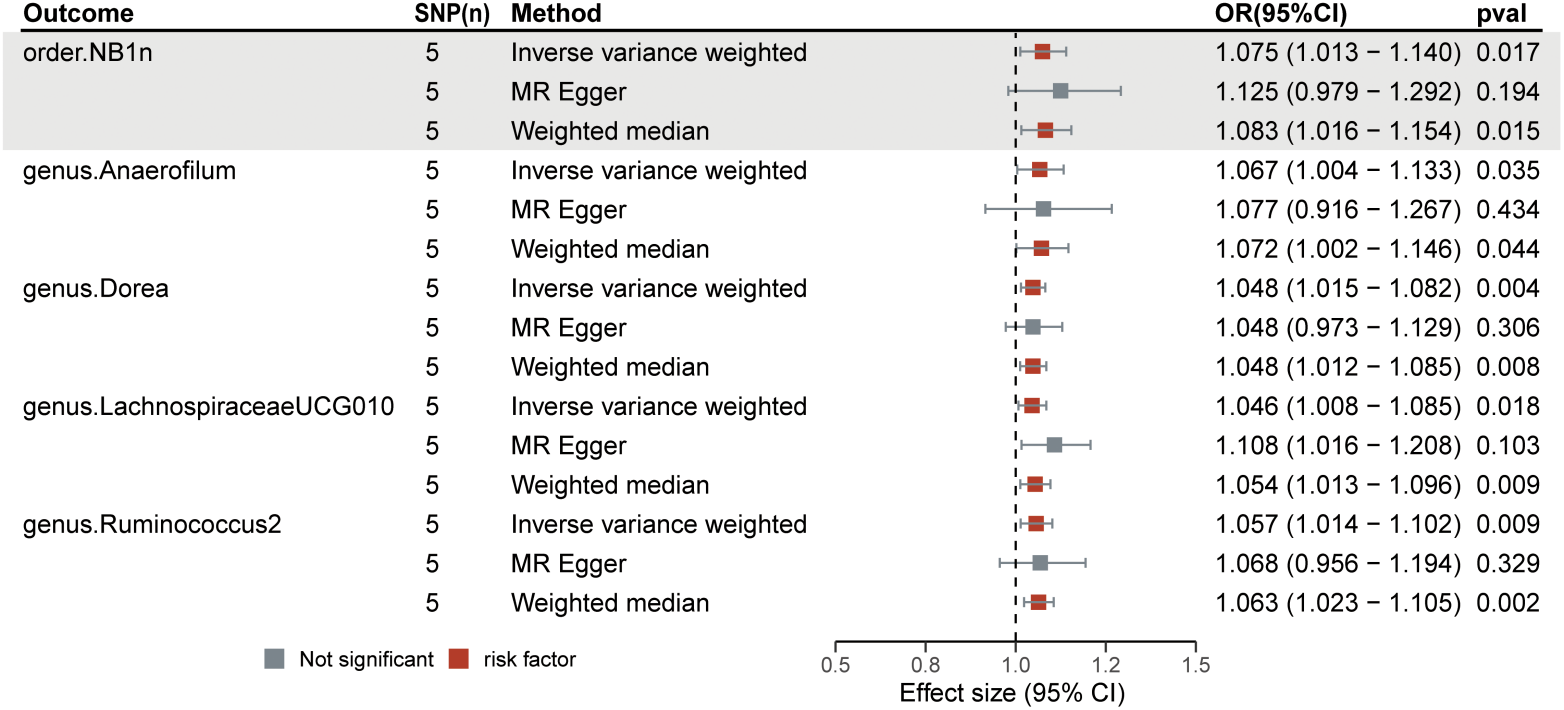
Forest plots of the significant causal effect of diabetic neuropathy on gut microbiota were calculated using the IVW method, MR-Egger, and weighted median. The forest plots demonstrate that diabetic neuropathy was a risk factor for the *Anaerofilum* (OR = 1.07; 95% CI = 1.00–1.13; *p*< 0.05), *Dorea* (OR = 1.05; 95% CI = 1.02–1.08; *p*< 0.01), *Lachnospiraceae UCG-010* (OR = 1.05; 95% CI = 1.01–1.09; *p =* 0.02), *Ruminococcus 2* (OR = 1.06; 95% CI = 1.01–1.10; *p* = 0.01), and order NB1n (OR = 1.08; 95% CI = 1.01–1.14; *p* = 0.02).

Among the 211 bacterial traits, five exhibited elevated levels that could be associated with an increased risk of DN. Details and statistics are given in **Figure 4**. These include the genus *Anaerofilum* (OR = 1.07; 95% CI = 1.00–1.13; *p*< 0.05), *Dorea* (OR = 1.05; 95% CI = 1.02–1.08; *p*< 0.01), *Lachnospiraceae* UCG-010 (OR = 1.05; 95% CI = 1.01–1.09; *p* = 0.02), *Ruminococcus 2* (OR = 1.06; 95% CI = 1.01–1.10; *p* = 0.01), and the order. NB1n (OR = 1.08; 95% CI = 1.01–1.14; *p* = 0.02). Forest plots were drawn using IVW, MR-Egger, and WMo (**Figure 5**). Next, sensitivity analysis of the MR results between DN and the five GMs (**Supplementary Table 4**) was performed, and the test showed no heterogeneity or horizontal pleiotropy. Details of IVs for reverse MR are listed in **Table 2**. The intercepts of the MR-Egger regression demonstrated no evidence of horizontal pleiotropy, as shown by *p* value > 0.05. The MR-PRESSO global test score of *p* > 0.05 indicated that there is no evidence of horizontal pleiotropy (**Supplementary Table 5**). Scatterplots (**Supplementary Figure 3**) and leave-one-out plots (**Supplementary Figure 4**) revealed no outliers.

**Table 2:** Instrumental variables used in MR analysis of the association between Diabetic neuropathy and gut microbiota.

Exposure	SNP	chr.	pos.	Beta	SE	*p*-value	R^2^	F
**Diabetic neuropathy**	rs13212435	6	32454571	-0.273	0.034	1.21787E-15	2.33e-04	64.0
	rs2476601	1	113834946	-0.274	0.036	5.60919E-14	2.06e-04	56.5
	rs2736428	6	31876147	0.195	0.029	1.78115E-11	1.65e-04	45.2
	rs73410776	6	32822178	0.525	0.04	4.55198E-40	6.39e-04	175.5
	rs9273364	6	32658525	0.593	0.027	5.7148E-105	1.72e-03	473.4

### 3.5 GO enrichment analysis

**Figure 6:**
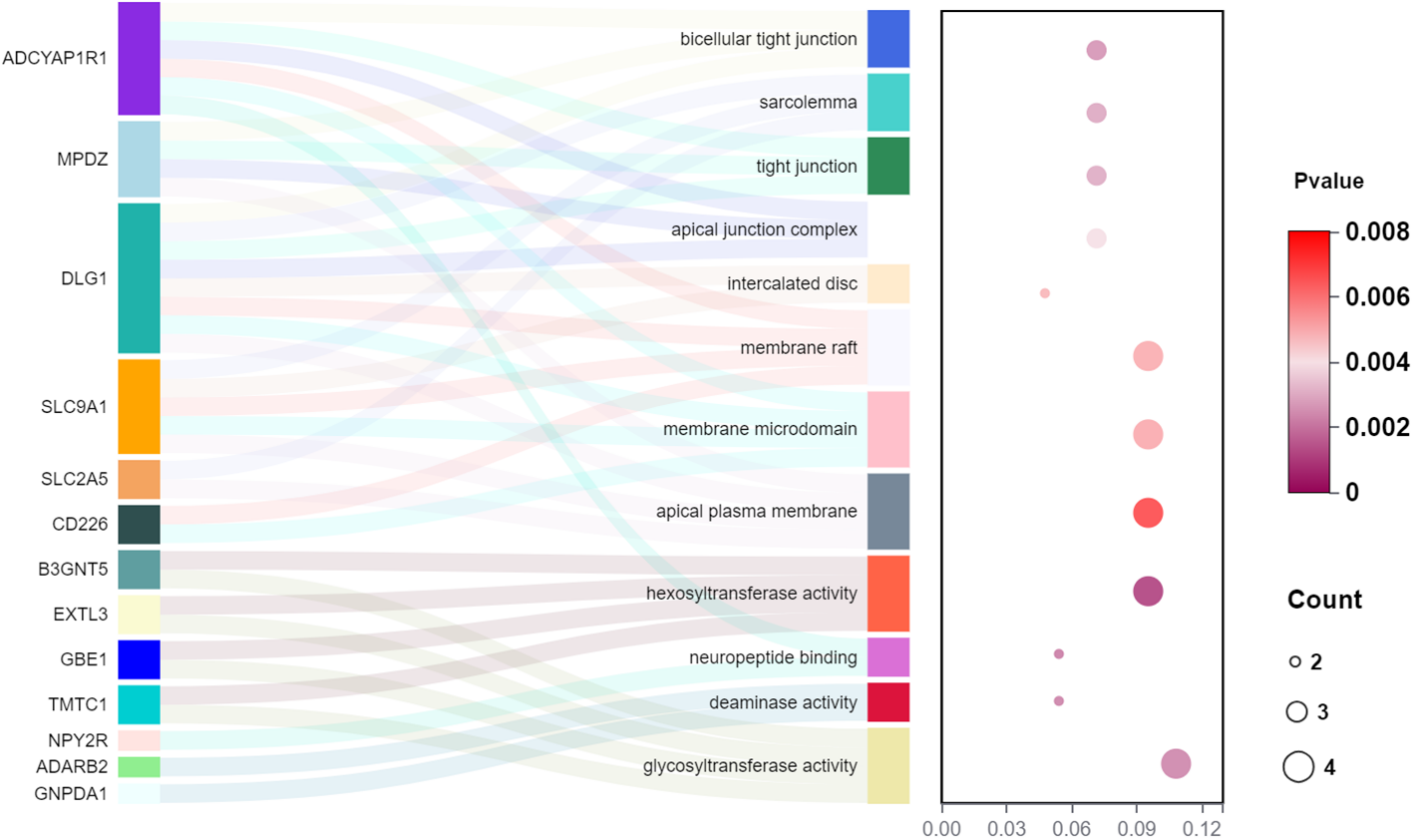
The GO enrichment analysis of the 13 genes from a cis-eQTL perspective in the forward MR analysis (gut microbiota as exposure and diabetic neuropathy as outcome).

In the set of IVs from the forward MR analysis, a total of 13 genes were identified: *ADCYAP1R1, MPDZ, DLG1, SLC9A1, SLC2A5, CD226, B3GNT5, EXTL3, GBE1, TMTC1, NPY2R, ADARB2*, and *GNPDA1*, which exhibited cis-regulatory control over gene expression. The GO enrichment analysis of these 13 genes yielded 12 significant results (*p*< 0.05), such as apical plasma membrane, glycosyltransferase activity, hexosyltransferase activity, and membrane raft (**Figure 6**).

## 4 Discussion

In recent times, there has been significant focus on the gut microbiota in metabolic illnesses, specifically type 2 diabetes. This topic has been extensively explored in scientific literature (28). In addition, previous studies have used MR analysis to elucidate potential causal relationship between various biomarkers from different sources and the risk of various diseases (29–31). In this study, we utilized GM data derived from a GWAS meta-analysis conducted by the MiBioGen consortium and DN data from the R8 release of the FinnGen consortium. The causal effects of GM taxa (from phylum to genus level) on DN were investigated. We found increased levels of Christensenellaceae R-7, Ruminococcaceae UCG013 and *Eggerthella* groups, which may be associated with a higher risk of DN, while increased levels of Peptococcaceae and *Eubacterium coprostanoligenes* groups could be linked to a lower risk. Additionally, we performed a reverse MR analysis to demonstrate the causal relationships between DN and GM, and found that the risk of DN may be potentially linked with elevated levels of *Anaerofilum, Dorea, Lachnospiraceae UCG-010, Ruminococcus_2*, and order NB1n. The GO enrichment analysis showed a considerable enrichment of the genes involved in glycosyltransferase and hexosyltransferase activities.

There is an increasing interest in studying the harmful effects of the microbiome on various human diseases. Dysbiosis of GM may disrupt normal gut microbial activity, leading to various neurological defects (32). Similarly, previous studies *in vivo* have shown that transplanting dysbiotic GM from individuals with distal symmetric polyneuropathy, a prevalent neuropathy in people with diabetes mellitus, to *db/db* mice had accelerated the development of peripheral neuropathy (33). *Ruminococcus* has been reclassified as *Blautia*, a genus of anaerobic bacteria that play specific roles in metabolic disorders, inflammatory diseases, and biotransformation (34). Recent investigations have shown that *Ruminococcus torques* level was significantly elevated in the clinically diagnosed DPN group, relative to the normal or disease controls (35). Similarly, Ruminococcaceae_UCG013 may be a causative agent of DN in our study. A prospective cohort analysis showed that *Eggerthella* is an important risk factor for diabetic foot ulcers (36), whereas peripheral neuropathy was identified as one of the most prominent variables linked with diabetic foot ulcers (37). implying that *Eggerthella* may be a potential risk factor for DN. These results were consistent with our study. Previous studies have confirmed that Peptococcaceae is a protective factor for diabetic retinopathy (38). However, the role of Peptococcaceae in DN has not been previously investigated. Besides, the reverse MR analysis suggested that DN may have a causal association with the elevated levels of *Ruminococcus_2.* There may be a two-way causal relationship between different genera of GM and the same disease. Therefore, further investigations are needed to clarify the functional significance of specific genera of GM and explore targeted therapies for gut bacterial flora. Previous research has shown a potential genetic relationship between the Christensenellaceae R-7 group and frailty, highlighting the significance of GM in human physiology (39). Our research confirms that Christensenellaceae R-7group has been associated with an increased incidence of DN, offering a novel avenue to explore the impact of GM.

There is less research on the effect of DN on GM. GM is dynamic and mostly stable in healthy people, but it can be influenced by many disorders (40). From the perspective of metabolic and immune processes, the distribution of GM significantly differed between the patients with and without diabetes (41). In our study, increased levels of *Anaerofilum*, *Dorea*, *Lachnospiraceae UCG-010*, *Ruminococcus_2*, and order NB1n may potentially be associated with the risk of DN. Previous studies have shown that delayed neurocognitive recovery was enriched by *Anaerofilum* compared to the non-delayed neurocognitive recovery group (42). The study of the relationship between *Dorea* and insomnia (43), *Lachnospiraceae UCG-010* and chronic kidney disease (44), *Ruminococcus_2* and rheumatoid arthritis (45), order NB1n and gastroduodenal ulcers (46) highlight the potential for GM-focused treatments.

The precise processes through which gut bacteria influence the likelihood of developing metabolic diseases are still unknown. Prior research has shown specific factors contributing to the progression of diabetic issues are elevated levels of reactive oxygen species, chronic hyperglycemia, reduced antioxidant capacity (47) and the anti-inflammatory effects of certain bacteria (such as, *Faecalibacterium* in patients with DN) (48). Moreover, numerous recent studies have recognized the gut–brain axis as a pivotal mechanism for investigating the advancement of diseases. However, there is a lack of research on the connection between the microbiota and various types of pain that lack a clearly identifiable localized cause, such as DN (49, 50). In our study, we performed a GO analysis for the 13 genes to find potential mechanisms of disease pathogenesis. The analysis showed an increase in the apical plasma membrane, glycosyltransferase activity, hexosyltransferase activity, and membrane raft.

This research has several strengths. Causal inference between GM and DN was determined using an MR analysis to exclude the confounding variables and reverse causation. The genetic variations of GM were derived from the most extensive GWAS meta-analysis to ensure that the robustness of the instruments used in the MR analysis. Horizontal pleiotropy was identified and ruled out by the MR-PRESSO and MR-Egger regression intercept term analyses. The leave-one-out analysis confirmed the robustness of the results. A two-sample MR test was used, utilizing non-overlapping exposure and result summary-level data to prevent bias (51). Nevertheless, our research has some drawbacks. We chose SNPs with *p*< 1 × 10^−5^ as IVs due to the limited number of SNPs with *p*< 5 × 10^−8^. We conducted multiple IV screening processes to ensure the reliability of IVs. This included removing SNPs with an F-value< 10 to prevent bias from weak IVs and scanning all SNPs in PhenoScanner V2 to eliminate any confounding effects. This study examines the relationship between GM and DN without studying the underlying mechanism. This MR analysis can be affected by potential pleiotropy. Each exposure in our study had a minimum of three IVs, which could potentially reduce the impact of pleiotropy given that distinct IVs are unlikely to exhibit the same correlation due to pleiotropy. The genetic IVs showed a slight effect on the variances of certain microbial taxa, possibly limiting the statistical power of the association findings.

The research participants were mostly of European descent, with limited GM data collected from other ethnic groups, who were less influenced by ethnic bias. Hence, this prevents the generalizability of the findings to other groups. Therefore, future research should examine the complex interactions and communications between the host and gut bacteria to enhance our understanding of the relation between GM and illness.

## 5 Conclusion

In conclusion, by carrying out a two-sample MR analysis using publicly available GWAS summary-level data, we investigated the causal influence of GM on DN neuropathy and found potential flora for DN development. This work may be relevant for screening gut microbial-derived metabolites and indicators for early diagnosis of DN, which could serve as non-invasive diagnostic or therapeutic targets.

## Data availability statement

The original contributions presented in the study are included in the article/**Supplementary Material**. Further inquiries can be directed to the corresponding author/s.

## Author contributions

LX: Conceptualization, Data curation, Formal analysis, Project administration, Writing – original draft, Writing – review & editing. WG: Data curation, Formal analysis, Methodology, Project administration, Supervision, Validation, Writing – review & editing. GC: Formal analysis, Funding acquisition, Project administration, Resources, Validation, Visualization, Writing – review & editing.
